# Comparisons of Pacing Strategy and Technical-Tactical Behaviors in Female Mixed Martial Arts Rounds

**DOI:** 10.3389/fpsyg.2020.548546

**Published:** 2021-01-29

**Authors:** Bianca Miarka, Gustavo Nascimento de Carvalho, Diego Ignácio Valenzuela Pérez, Esteban Aedo-Muñoz, Ciro José Brito

**Affiliations:** ^1^Postgraduate Program in Physical Education, Department of Fights, School of Physical Education and Sports, Federal University of Rio de Janeiro, Rio de Janeiro, Brazil; ^2^Escuela de Kinesiologia, Universidad Santo Tomas, Santiago, Chile; ^3^Biomechanics Laboratory, Physical Activity, Sport and Health Sciences Laboratory, Chilean High Performance Center, Universidad de Santiago de Chile, Santiago, Chile; ^4^Postgraduate Program in Physical Education, School of Physical Education and Sports, Federal University of Juiz de Fora, Juiz de Fora, Brazil

**Keywords:** sport psychology, decision-making, technical- tactical analysis, gender, time-motion analysis

## Introduction

Socially and historically, there has been a clear distinction between combat sports experienced by men and women, and only in the last two decades have better chances and recognition for women become apparent (Kociuba et al., 2017). For instance, in the United States, prior to the success of the female Ultimate Fighter Championship, there was little primary coverage of female combat competitors (Miarka et al., 2016). Official female mixed martial (MMA) events usually consist of three to five 5-min rounds that can be won by referee's decision (Matthews and Nicholas, 2016) and that can be decided before the end of the official combat time, modifying fighting and preparatory times on standing and ground combat (Antonietto et al., 2019). This fight-ending or ending-round has a winning criterion, involving a loss of consciousness called knockout (KO) or a technical knockout (TKO) when the referee decides during a round that an MMA athlete cannot safely continue the combat for any reason (Follmer et al., 2019). These ending-rounds by KO or TKO could occur from an intense strike sequence or with a precise single strike action (James et al., 2017) or by a choke or joint locking submission (James et al., 2017).

During MMA combat, the athletes usually begin combat trying to gain an advantage through strike attacks during the standing preparatory-activity time (i.e., displacements or stable positions and movements without opposition or with isolated attack) (James et al., 2016). Next, come attempt punches, kicks, and takedowns followed by ground combat with striking and grappling techniques (James et al., 2017). The strategic distribution of fighting time, which includes numerous offensive and defensive techniques, exchanges between opponents, and preparatory time by round, can provide a tactical advantage over rounds and better attack selection (Antonietto et al., 2019), and it is associated with a possible consequent reaction by the opponent (Seniuk et al., 2019). Information about KO and TKO effects in female MMA combat could improve practical application, as the knowledge margin of victory relevantly affects technical and tactical aspects in situational sports (Lupo and Tessitore, 2016). Furthermore, in high-level MMA, technical-tactical behaviors rely on anticipation of subsequent events as well as concurrent and quick responses to spatiotemporal changes (Smits et al., 2014). Therefore, the frequency and duration of specific behaviors during end-points in female MMA combat and the physical sensations of effort and fatigue are all associated with adequate timing and perception to effectively execute a behavior (Antonietto et al., 2019).

To maximize performance, MMA coaches have to make strategic decisions about how their female athlete should invest their energy during the rounds and combat (James et al., 2016). Due to difficulties in conducting physiological measurements during combats championships, researchers interested in the match demands have used combat phase analysis of the preparatory-activity with low intensities and fighting-activity and with high intensities to determine the metabolic profile (Chaabene et al., 2014; Menescardi et al., 2015; Slimani et al., 2017). Nevertheless, to obtain information for conditioning and strength training of athletes, it is significant to highlight that open task and intermittent combat sports involve complex sequential skills (Dos Santos et al., 2019; Ghoul et al., 2019; Vasconcelos et al., 2020), with a range of around 92.2 ± 71.0–162.3 ± 89.4 s of preparatory-activity with low-intensity actions to 33.5 ± 52.2–69.8 ± 79.6 s of fighting activity with high-intensity actions (Dos Santos et al., 2019).

This concept is also known as pacing strategy (Smits et al., 2014) and, this phenomenon has not been investigated in female MMA combat. A full literature review did not result in any research addressing the capability to integrate technical-tactical behaviors and pacing strategy based on reactive/proactive dynamic behaviors during rounds, with female combat ending in a KO or effective submission. Based on this knowledge, present research used a well-established investigational paradigm to study pacing specific to female MMA phases during sequential behaviors, which required an extensive database to demonstrate sequential behaviors (Smits et al., 2014). This study provides an overview of pacing strategies by the outcome, which is called the ending-round in the first round vs. second round vs. third round. The current analysis is essential to recognize essential mechanisms that are important and their intensity. The present study thus aimed to (i) typify the performance effects during continuous self-paced sequential female MMA rounds, (ii) verify the round effects in time-motion results of female MMA athletes during sequential combat phases, and (iii) verify the technical-tactical behaviors attempted and landed during each ending-round and round.

## Methods

### Sample

The sample was composed of 13,572 sequential behavior analysis from 74 different female MMA fights that ended in KO or TKO. It included 174 rounds, separated into the round (1Rx1ER) of combat ending in the first round (*n* = 22); first round (1Rx2ER) and second round (2Rx2ER) of combat ending in the second round (*n* = 28); and first round (1Rx3ER), the second round (2Rx3ER), and third round (3Rx3ER) of combat ending in the third round (*n* = 124) from matches between 2014 and 2018 from all weight divisions from the following events: UFC 220, UFC 221, UFC Fight Night 124, UFC on Fox 28, UFC 228, UFC 223, UFC on FOX 29, UFC 225, UFC 224, UFC 226, UFC Fight Night 126, UFC on FOX 30, UFC Fight Night 125, Bellator 221, UFC Fight Night 129, ONE FC, Invicta FC 30, Invicta FC 28, Invicta FC 27, and Invicta FC 32. We estimated the Finite Population sample size, considering a 99.9% confidence level with a 5% margin of error from the current pound-for-pound female fighter ranking and the number of female combats in UFC events/year. Each athlete had a minimum of 6 weeks of rest from her previous combat to prevent stress interference. All participants had previous experience with professional MMA events, rules, and procedures. No modifications were made in the training, nutritional, or hydration status of participants, and they maintained the weight loss recovery time pattern of 24 h between the Official weigh-in and the combat following MMA rules (Matthews and Nicholas, 2016).

The inclusion criteria considered only scheduled three-combats (including KO/TKO and submission combat endings), while exclusion criteria were concerning combats with referee's decision or with more than three rounds or with characteristics that disqualified prospective outcomes comparisons—combats that finished in “draw” or “no contest.” The present study is a post facto study; even so, the present investigation was submitted to and approved by the Health, Medical & Research Committee of Ethics (051979-2017). All fighters were >18 years old and signed an informed consent document to understand the benefits and risks of participating in UFC bouts, ensuring anonymity, and following the Declaration of Helsinki.

### Behaviors and Performance Analysis

Sequential behavior analysis was observed according to each frequency of occurrence and time and then showed by round pacing, defined by the moment of each combat phase (Antonietto et al., 2019):

Standing preparatory-activity—Standing up combat, which consists of displacements or stable positions and actions without opposition or an isolated attack (Chaabene et al., 2014; Slimani et al., 2017).

Standing fighting-activity—Standing up combat, which consists of multiple offensive and defensive techniques, exchanges between adversaries (Chaabene et al., 2014; Slimani et al., 2017).

Ground preparatory-activity—Groundwork combat, which consists of stable positions and actions without opposition or an isolated attack (Chaabene et al., 2014; Slimani et al., 2017).

Ground fighting-activity—Groundwork combat, which consists of multiple offensive and defensive techniques, exchanges between adversaries (Chaabene et al., 2014; Slimani et al., 2017).

Concerning technical-tactical behavior analysis during the round, the present study followed previously published research protocols that define the following:

(a) Strike behaviors—any attempt to carry out striking actions (punches, kicks, elbows, and knees), separated by the targets (i.e., opponent's head, body, or leg) (James et al., 2017; Fernandes et al., 2018; Aedo-Muñoz et al., 2019).
(b) Takedowns—technical-tactical behavior that includes off-balancing and attempts to take the opponent off their feet, typically with the attacker landing on top (Kirk et al., 2015; Fernandes et al., 2018; Aedo-Muñoz et al., 2019).
(c) Submission attempts—when one of the fighters tries to control the opponent on the ground (Kirk et al., 2015; Fernandes et al., 2018; Aedo-Muñoz et al., 2019).
(d) Locks—single or double joint lock including management of an opponent's joints in such a way that the joints arrive at their maximal degree of motion (Kirk et al., 2015; Fernandes et al., 2018; Aedo-Muñoz et al., 2019).
(e) Chokes—consist of an attempt to mechanically stop the flow of air toward the lungs of the opponent or applying pressure to the central nervous, vascular system (carotid arteries) (Bello et al., 2019).

The reliability measures were assessed through intra-observer and inter-observer testing procedures, with an agreement classified as “Substantial” or “Almost Perfect” for all time-motion variables, with an agreement of 0.98 and 1.00 for standing preparatory-activity, 0.99 and 0.99 for standing fighting-activity, 0.98 and 0.99 ground preparatory-activity, and 0.99 and 0.99 ground fighting-activity, respectively. Regarding technical-tactical analysis, the agreement from intra-observer and inter-observer were 0.82 and 0.85 for strike attempts, 0.85 and 0.95 for takedown attempts, and 0.80 and 0.94 for submission, respectively.

### Statistical Analysis

The normal distribution of data was observed using the Kolmogorov-Smirnov test. Descriptive data of continuous time-motion variables are presented as mean and standard deviation (SD), while frequencies are presented as median, first quartile (1Q), and third quartile (3Q). Two-way [finished Round (first round, second round, and third-round) × Round (first round, second round, and third-round)] (ANOVA) with Bonferroni *post-hoc* tests were used to compare the combat phases, and eta-squared was informed. The variance analysis (ANOVA) was used to estimate our continuous time-motion round and ending-round means and the differences in the round means and ending-round means. Two-way ANOVA tests the hypotheses for statistical significance of comparisons of round and ending-round means, where round and ending-round characteristics distinguish the time-motion variables of female MMA combats. For the non-parametric data, Kruskal Wallis's analysis and Dunn's *post-hoc* test were applied to compare round vs. round. Kruskal Wallis was used for comparing the rounds; since this test is a non-parametric method, our technical-tactical behavior data did not assume a normal distribution, unlike the analogous ANOVA. The significance level used was *p* ≤ 0.05, and the Statistical Package for the Social Sciences (SPSS version 20.0, Chicago, USA) was used to conduct the analysis.

## Results

Table 1 showed the descriptive analysis of dynamics states of continuous-time in each round phase in standing (preparatory and fighting) and ground behaviors (preparatory and fighting).

**Table 1:** Descriptive analysis of continuous time in each round phase of female professional MMA combats in standing (preparatory and fighting) and ground actions (preparatory and fighting).

**Round**	**Ending round**	**Standing preparatory-activity**	**Standing fighting-activity**	**Ground preparatory-activity**	**Ground fighting-activity**
		**M ± SD**	**M ± SD**	**M ± SD**	**M ± SD**
1R	1ER	34.09 ± 39.94	7.41 ± 14.52	0.00 ± 0.00	1:03.91 ± 1:26.99
	2ER	31.00 ± 12.70	16.25 ± 32.50	0.00 ± 0.00	1:31.75 ± 1:20.44
	3ER	2:32.42 ± 1:33.08	24.73 ± 42.25	0.00 ± 0.00	41.46 ± 1:16.29
	Total	1:50.68 ± 1:36.46	19.12 ± 36.27	0.00 ± 0.00	50.85 ± 1:19.94
2R	2ER	1:03.00 ± 45.03	3.25 ± 6.50	0.00 ± 0.00	53.50 ± 1:47.00
	3ER	2:32.17 ± 1:27.98	18.08 ± 28.27	0.21 ± 1.01	51.46 ± 1:16.40
	Total	2:25.31 ± 1:28.48	16.94 ± 27.48	0.19 ± 0.97	51.62 ± 1:17.80
3R	3ER	2:26.92 ± 1:25.55	14.96 ± 26.03	0.58 ± 2.82	46.79 ± 1:11.11
	Total	2:26.92 ± 1:25.55^@^	14.96 ± 26.03	0.58 ± 2.82	46.79 ± 1:11.11
Total	1ER	34.09 ± 39.94	7.41 ± 14.52	0.00 ± 0.00	1:03.91 ± 1:26.99
	2ER	47.00 ± 35.08	9.75 ± 22.78	0.00 ± 0.00	1:12.62 ± 1:29.99
	3ER	2:30.50 ± 1:28.34	19.26 ± 33.01	0.26 ± 1.73	46.57 ± 1:14.23
	Total	2:11.02 ± 1:32.36	17.32 ± 31.06	0.22 ± 1.58	49.96 ± 1:16.54

*M, mean; SD, standard deviation; R, round; ER, ending round; Time: min:s.ms*;

@ *, significant difference when compared with total of 1ER, p ≤ 0.05*.

**Figure 1:**
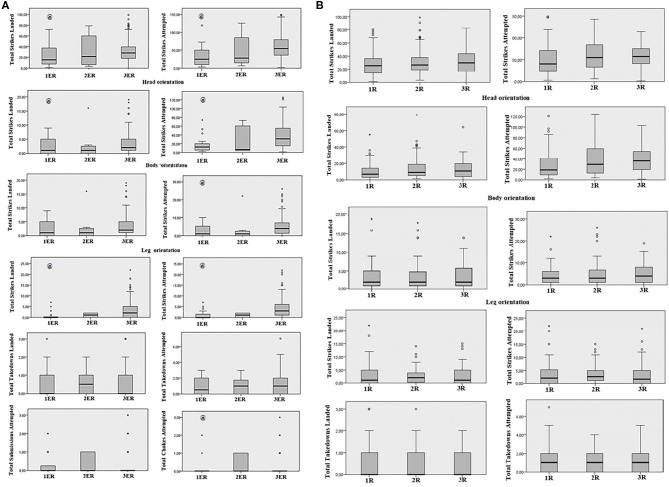
Technical-tactical behavior analysis of female MMA rounds ratios. **(A)** Technical and tactical behavior attempted; **(B)** Technical and tactical behavior landed. R, round; Locks, chokes, and submission attempts = 0.0 and no effects were observed (*p* > 0.05 for all comparisons).

Significant differences were observed between moments when compared standing preparatory-activity between rounds and ending-rounds (*F*_5, 173_ = 9.33, *p* ≤ 0.001, η^2^ = 0.22). The 3ER had a shorter time than 1ER (*p* ≤ 0.001). Analysis indicated differences between moments when compared standing preparatory-activity between ending-rounds (*F*_5, 173_ = 7.31, *p* ≤ 0.001, η^2^ = 0.08): the 3ER had shorter time than 1ER (*p* ≤ 0.001) and 2ER (*p* = 0.002). Figure 1 and Table 2 show the descriptive analysis of technical-tactical action frequencies by ending-round and demonstrates the analysis of technical-tactical action frequencies by a round of female professional MMA combats.

**Table 2:** Technical-tactical behavior analysis of female MMA rounds by frequencies (Median−50th, first quartile−25th and third quartile−75th, respectively), separated by rounds and ending-rounds.

	**1ER**	**2Rx3ER**	**1Rx3ER**	**2ER**	**2Rx3ER**	**3ER**
**Variables**	**50th (25th; 75th)**	**50th (25th; 75th)**	**50th (25th; 75th)**	**50th (25th; 75th)**	**50th (25th; 75th)**	**50th (25^**th**^; 75th)**
**Landed actions**						
Total strikes	15.5(7.5; 37.3)	21.5(11.8; 33.5)	28.0(16.3; 39.8)	36.5(3.5; 76.3)	26.5(20.3; 36.5)	30.0(16.3; 43.5)^@^
Head strikes	5.5(2.0; 10.0)	3.0(2.3; 3.8)	10.0(4.0; 15.5)	23.0(2.0; 45.5)	9.0(5.0; 17.8)	10.5(3.5; 19.8)^@^
Body strikes	1.0(0.0; 5.0)	0.5(0.0; 2.5)	2.0(1.0; 5.0)	1.0(0.3; 12.3)	2.0(1.0; 4.8)	2.0(1.0; 5.8)
Leg strikes	0.0(0.0; 0.3)	1.5(0.3; 2.0)	2.0(1.0; 6.0)	0.5(0.0; 1.8)	2.0(1.0; 4.0)	1.0(0.0; 5.0)^@^
Takedowns	0.0(0.0; 1.0)	1.0(0.3; 1.8)	0.0(0.0; 0.0)	0.0(0.0; 0.8)	0.0(0.0; 1.0)	0.0(0.0; 1.0)
**Attempted actions**						
Total strikes	24.5(9.8; 50.5)	25.0(15.0; 38.0)	49.0(34.3; 81.0)	61.5(10.3; 119.5)	55.5(34.5; 82.3)	57.5(41.5; 75.3)^@^
Head strikes	12.0(5.5; 19.8)	5.5(4.3; 6.8)	26.5(15.5; 50.0)	48.0(8.8; 73.0)	29.0(12.5; 57.0)	36.0(19.0; 54.3)^@^
Body strikes	1.0(0.0; 5.3)	0.5(0.0; 2.5)	4.0(2.0; 7.0)	1.0(0.3; 16.8)	3.5(1.3; 6.8)	4.0(1.0; 8.0)
Leg strikes	0.0(0.0; 1.5)	1.5(0.3; 2.0)	3.0(1.0; 7.0)	0.5(0.0; 1.8)	3.0(1.0; 5.0)	1.5(0.0; 5.0)^@^
Takedowns	0.5(0.0; 2.0)	1.5(0.3; 2.8)	1.0(0.0; 2.0)	0.5(0.0; 1.0)	1.0(0.0; 2.0)	1.0(0.0; 2.0)
Submission	0.0(0.0; 0.3)	0.5(0.0; 1.0)	0.0(0.0; 0.0)	0.0(0.0; 0.8)	0.0(0.0; 0.0)	0.0(0.0; 0.0)
Chokes	0.0(0.0; 0.0)	0.5(0.0; 1.0)	0.0(0.0; 0.0)	0.0(0.0; 0.8)	0.0(0.0; 0.0)	0.0(0.0; 0.0)^#^

*R, round; ER, ending-rounds; Lock attempts had all quartiles (50th; 25th; and 75th) <0.02 and no effects were observed between them*.

@ *Significant difference when compared with 1ER*;

# *Significant difference when compared with ER p ≤ 0.05*.

A significant main effect was observed when comparing total strikes: landed (*X*^2^ = 7.15, df = 2, *p* = 0.028), landed on the head (*X*^2^ = 6.51, df = 2, *p* = 0.039), and total attempts on the head (*X*^2^ = 16.91, df = 2, *p* ≤ 0.001); all *post-hoc* showed a higher frequency in the 3ER than 1ER (*p* = 0.027 for landed; *p* = 0.05 for landed on the head and; *p* ≤ 0.001 for attempts on the head). For body strikes, no effects were observed when compared strikes landed (*p* ≥ 0.05 for all comparisons). However, a significant difference was verified when compared total strikes attempted (*X*^2^ = 9.75, df = 2, *p* = 0.008); 3ER was higher than 1ER (*p* = 0.043).

For leg strikes, differences were observed between ending-rounds when compared the strikes landed (*X*^2^ = 18.93, df = 2, *p* ≤ 0.001) and total strikes attempts (*X*^2^ = 16.79, df = 2, *p* ≤ 0.001), where 3ER was higher than 1ER (*p* ≤ 0.001 for all comparisons). When compared chokes attempts (*X*^2^ = 7.56, df = 2, *p* = 0.023), 2ER was higher than 3ER (*p* = 0.018). No effects were observed between rounds without ending-round (*p* ≥ 0.05 for all comparisons).

## Discussion

The present research sought to understand the mechanisms involved in pacing strategy and technical-tactical behaviors used in the reactive/proactive dynamics by a round of female professional MMA combat. The main results indicated an increase in the frequency of attacks to the head, body, leg orientations, and the preparatory activity time compared to 1ER vs. 3ER. The correlation of round phases during MMA combats and critical technical-tactical behaviors, such as takedowns, submissions, and strike attempts in different orientations (i.e., to the head, body, or leg), represent the main aspects of attacking systems used by women. Besides, to obtain information for conditioning and strength training of female athletes, it is significant to highlight that open task and intermittent combat sports involve complex sequential skills, with a range of around 92.2 ± 71.0–162.3 ± 89.4 s of low-intensity behaviors to 33.5 ± 52.2–69.8 ± 79.6 s by high intensity.

Investigations in female MMA intensity are rare, but this kind of study could help improve training by identifying specific movements and specific-intensity ratio simulations (Smits et al., 2014). Many studies have revealed how reactive/proactive, dynamic performances, and pacing are connected (Smits et al., 2014), and it has been established that male athletes tend to self-select strategies and technical-tactical behaviors (Antonietto et al., 2019) closely associated with their psycho-physiological disposition throughout the natural interactive performance. Furthermore, approaches to pacing and technical-tactical behaviors changed when the female athletes were strategically in two situations (i) in an attempt to cause TKO/KO in the first, second, or third round and, (ii) when the athletes are in combat with the relatively equal opponent and finish the combat during the third round. Present results without differences between rounds demonstrated that the time of combat phases and technical-tactical frequencies between female MMA athletes are very similar (Tables 1, 2). In contrast, the frequency of the behaviors exhibited differences when they occurred intending to finish the combat, particularly in the last round.

Present results indicated that athletes aimed to increase the total attacks of head, body, and leg orientations followed by choke attempts during combats with three rounds, especially using single action in preparatory activity time during the third ending-round. This observation has a physiological significance, as it indicates that the round intensity during combat phases was self-regulated relative to the female athlete's physical capability near the end of the combat. Additionally, previous research differentiated weight categories (Miarka et al., 2016), which could be considered in studying pacing strategies. However, it is essential to note that analysis of technical-tactical behaviors has limitations associated with the evaluator's ability to determine the technical-tactical action and (Fernandes et al., 2018; Dos Santos et al., 2019). Our research used experienced evaluators with more than 10 years of experience in the knowledge associated with MMA behaviors and time-motion analysis to minimize the bias-related to data collection. Our data indicated high reliability associated with intra-expert and inter- expert results, with a “Substantial” or “Almost Perfect” agreement for all time-motion and behavior analyses. Therefore, based on this research aims, we can indicate that pacing strategy and technical-tactical behaviors differ between female MMA ending-rounds.

The results of this research agree with previous research, which shows a more significant number of strikes attempted and landed in combat ending during the last round (Antonietto et al., 2019). However, different from male combats, female ending combat in the 1ER had a lower ratio of strike attempts than the 3ER, and different landed attacks with better effectiveness (i.e., considering landed by attempted attacks)−62% of strike attacks scored during the 1ER, 47% of effectiveness during the 2ER, while TKO/KO during the 3ER showed 52% of effectiveness attacks. Male combat demonstrated better effectiveness and higher strike attempts during the second and third rounds (Miarka et al., 2018). Specific contextual training programs with actual intensity for female TKO/KO situations are critical because if the match reaches its time limit, then the combat outcome is determined by the three judges. The UFC system is under a 10-point scoring system(Fernandes et al., 2018). During the present collection data, close rounds scored 9–10. When women overwhelmingly won combat, it was scored 8 to 10 points in the last round (UFC, 2019). The UFC score is based on landed striking actions, and this score is determined by how many legal strike attempts are landed, and present research followed this rule (Fernandes et al., 2018). The MMA fight is scored in its entirety and not round-by-round, which makes the MMA pacing strategy per round essential.

Furthermore, the current ratios agree with previous studies with male athletes that showed intensity ratios were similar between all rounds, about 1:2 to 1:4 of high: low-intensity time (Antonietto et al., 2019). These results support our thought that an efficient strategic approach to achieve the TKO/KO finish was applied by winners in UFC combats without TKO/KO (Miarka et al., 2016). A previous study indicated lower strike attempts of female combats than our results, with 33.9 ± 27.9 in the 1st round and higher values in the third Rounds, with 49 ± 31.6 attempts per round (Miarka et al., 2016). The present research found 40.5 (25; 71.5) strike attempts in the first round and 57.5 (41.5; 75.25) strike behaviors in the third round. These differences may be associated with the specialization of women, considering the time between studies and samples.

For groundwork activity and submission attempts, present female data showed higher choke attempts during the 2ER vs. 3ER. Summarizing, our data point to higher submission diversity in male combat and the tendency to apply choke techniques in the women. These results demonstrated higher strike attempts when compared with previous research that also showed significant differences between ending rounds, 26.1 ± 48.3 for first ending round, 25.0 ± 41.8 for second ending round, and 52.1 ± 73.7 for third ending round ground time with high intensity, respectively (Miarka et al., 2018). Previous studies with male athletes suggested that ~77% of combats were decided during fighting activity in standing combat or in-ground grappling sequences and grappling behaviors. Technical proficiency as the main factors that contribute to winning outcomes (Coswig et al., 2015), with a difference between winning and losing combats of 0.2 ± 1.0 in clinch strikes landed per min and 0.1 ± 0.3 in takedowns landed per min (James et al., 2017). The present research found values of 1.1 ± 1.3 takedowns and 0.4 ± 0.7 takedowns landed by female MMA round. This finding suggests that the development of grappling technical-tactical behaviors may enhance performance, especially during female MMA combat that is strategically prolonged to the last round to influence a decision. The practical application of these results may suggest round circuit training and evaluations of the ratio round, such as the example in Table 3.

**Table 3:**
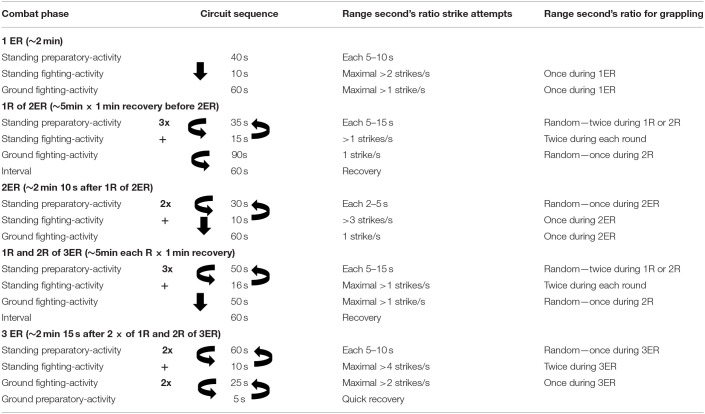
Example of practical application of the results associated with the combat phase and technical-tactical behaviors.

## Conclusion

The information generated from this research will help female MMA professionals create more effective mental and physical design training programs that improve performance. The pattern of strike pacing behaviors during female combats showed differences between the first and third ending rounds, with a pacing-round of ~0.5 strike attempts by second of fighting activity time in the first round by second of fighting activity time and ~1.0 strike attempts by second of fighting activity time in the second and third round. According to rounds, we suggest specific contextual training as if the female athlete intends to conduct the combat for all rounds. It is essential to increase strike frequency, from ~40 attempts, in the first round, to ~55–60 punches and kicks during the second and third ending-rounds. Results could suggest isolated training of each situation 1ER, 2ER, and 3ER based on each contextual situation's physiological demands, as demonstrated in Table 3. The competitive strategy, of course, must also account for achieving technical proficiency in defending strikes and takedown attempts, as well as grappling behaviors, especially during the second round of ground fighting activity.

## Data Availability Statement

The original contributions presented in the study are publicly available. This data can be found at: https://drive.google.com/open?id=15CtU1GySnC0-uKei9n9DA9KWurMS5d6D.

## Ethics Statement

The studies involving human participants were reviewed and approved by University of Rio de Janeiro CAAE: 23778719.7.0000.5257. The patients/participants provided their written informed consent to participate in this study. Written informed consent was obtained from the individual(s) for the publication of any potentially identifiable images or data included in this article.

## Author Contributions

All authors listed have made a substantial, direct and intellectual contribution to the work, and approved it for publication.

## Conflict of Interest

The authors declare that the research was conducted in the absence of any commercial or financial relationships that could be construed as a potential conflict of interest.
